# Risk of chronic traumatic encephalopathy in rugby union is associated with length of playing career

**DOI:** 10.1007/s00401-023-02644-3

**Published:** 2023-10-24

**Authors:** William Stewart, Michael E. Buckland, Bobak Abdolmohammadi, Andrew J. Affleck, Victor E. Alvarez, Shannon Gilchrist, Bertrand R. Huber, Edward B. Lee, Donald M. Lyall, Christopher J. Nowinski, Emma R. Russell, Thor D. Stein, Catherine M. Suter, Ann C. McKee

**Affiliations:** 1 School of Psychology and Neuroscience, University of Glasgow, Glasgow, G12 8QQ UK; 2 Department of Neuropathology, Queen Elizabeth University Hospital, 1345 Govan Rd, Glasgow, G51 4TF UK; 3 Department of Neuropathology, Royal Prince Alfred Hospital, Camperdown, NSW Australia; 4 School of Medical Sciences, University of Sydney, Camperdown, NSW Australia; 5 Alzheimer’s Disease Research Center and Chronic Traumatic Encephalopathy Center, Chobanian and Avedisian School of Medicine, Boston University, Boston, MA 02118 USA; 6 US Department of Veteran Affairs, Veterans Affairs (VA) Boston Healthcare System, Boston, MA 02130 USA; 7 Department of Neurology, Chobanian and Avedisian School of Medicine, Boston University, Boston, MA 02118 USA; 8 Department of Pathology and Laboratory Medicine, Chobanian and Avedisian School of Medicine, Boston University, Boston, MA 02118 USA; 9 US Department of Veteran Affairs, VA Bedford Healthcare System, Bedford, MA 01730 USA; 10 National Center for PTSD, VA Boston Healthcare, Boston, MA 02130 USA; 11 Translational Neuropathology Research Laboratory, University of Pennsylvania, Philadelphia, PA 19104 USA; 12 School of Health and Wellbeing, University of Glasgow, Glasgow, G12 8QQ UK; 13 Concussion Legacy Foundation, Boston, MA 02115 USA; 14 Department of Biostatistics, Boston University School of Public Health, Boston, MA 02118 USA

There is concern over late, adverse brain health outcomes associated with contact sports participation, with high neurodegenerative disease risk reported in studies of former American football [3, 8], soccer [9, 16] and rugby union players [15]. In parallel, autopsy studies of former athletes from a range of contact sports describe a frequent finding of chronic traumatic encephalopathy (CTE), a neuropathology uniquely associated with prior history of traumatic brain injury (TBI) and repetitive head impact (RHI) exposure [7, 12–14]. Among contact sports, rugby union (hereafter ‘rugby’) is documented as having high risk of concussion/mild TBI, with reported injury rates ranging 4.1 concussions/1000 player hours at community level [2] to 22.2 concussions/1000 player hours in professional rugby [4]. Nevertheless, despite its popularity, with a reported 8.46 million active participants globally [20], there have been relatively few case descriptions of CTE in former rugby players [7, 17, 18]. To address this, we collated and analyzed neuropathological data from autopsy brain examinations on individuals with rugby as primary sport exposure submitted to three international brain banks with specific interest in contact sport and brain health.

Case records of the Understanding Neurologic Injury and Traumatic Encephalopathy Brain Bank (UNITE; Boston University School of Medicine, US), the Glasgow TBI Archive (GTBI; University of Glasgow, UK) and the Australian Sports Brain Bank (ASBB; Royal Prince Alfred Hospital and University of Sydney, Australia) were surveyed to identify case donations in which primary sport exposure was recorded as ‘rugby union’. Each archive employs standardized procedures for case accrual, clinical history acquisition and tissue processing, with neuropathological evaluations conducted blind to demographic and clinical information and employing established, consensus protocols for assessment of neurodegenerative disease pathologies, including CTE [1, 11, 13]. For the purposes of this study, existing archive datasets were interrogated to extract relevant demographic information (age at death, sex), sports exposure history (years duration of rugby participation, position played [dichotomized as forward or back], highest level of participation [dichotomized as amateur or elite (encompassing representative international and/or professional)], other contact sport exposure) and principal neuropathological findings.

In total, 31 cases where primary sports exposure was documented as rugby union were identified within contributing research brain banks: 16 cases from UNITE; 8 from GTBI; and 7 from ASBB. Among these, mean age at death was 60.4 years (standard deviation [SD] 21.7 years; range 17–95 years), with all but 1 (3%) case male. Reported rugby career length averaged 18.3 years (SD 10.0 years; range 2–35 years), with an equal number of forward and backs, where information on player field position was available. Twenty-three (74%) played rugby exclusively as amateurs, with 8 (26%) reaching elite (representative/ professional) level and 19 of 29 (66%) reporting history of prior TBI with loss of consciousness and/or history of concussion.

Regarding neuropathological findings, CTE was present in 21 of 31 (68%) brains of former rugby players examined, a majority of whom (13/21; 62%) played solely at amateur level. Among cases with CTE, 14 were typical of low-stage CTE pathology, 7 high stage [1]. No notable differences were observed between players with or without CTE in respect of age at death, participation in other contact sports, history of drug or alcohol use disorder, whole brain weight at autopsy, prevalence of septum pellucidum abnormalities (Table 1) or wider neuropathologies assessed (Supplementary Table 1). However, players with CTE typically had longer rugby playing careers than those without CTE. Indeed, adjusted for age at death, a dose–response relationship was evident between career length and the presence of CTE at autopsy, with each additional year of play associated with an approximately 14% increase in CTE risk (relative risk ratio 1.138; 95% confidence interval 1.015–1.277; *P* = 0.027; multinomial logistic regression). While history of TBI with loss of consciousness and/or concussion was common among cases with CTE, this was not significantly different to prevalence among cases without CTE.

**Table 1:** Demographic and primary neuropathological findings

	CTE *N* = 21	No CTE *N* = 10	P
Demographic information			
Mean age at death	61.7 years	57.8 years	0.710*
(standard deviation) [range]	(17.5)[23–94]	(29.7)[17–95]	
Sex			NA
Male	21	9	
Female	0	1	
Years rugby participation	21.5 years	12.1 years	0.031*
(standard deviation) [range]	(8.1)[8–35}	(10.9)[2–15]	
Position			NA
Unknown	7	8	
Forward	7	1	
Back	7	1	
Highest participation level			NA
Amateur	13	10	
Elite	8	0	
Other contact sports			1.000**
None	12	6	
American football	5	2	
Boxing	3	0	
Soccer	2	1	
Ice Hockey	2	0	
Rugby league	1	0	
Wrestling	0	1	
TBI with LOC and/or concussion			0.083**
Unknown	0	2	
None	5	5	
Yes	16	3	
Alcohol use disorder			1.000**
Unknown	5	4	
No	9	4	
Yes	7	2	
Drug use disorder			0.120**
Unknown	6	4	
No	12	2	
Yes	3	4	
Neuropathology findings			
Mean brain weight	1353 g	1321 g	0.677*
(standard deviation)[range]	(208 g)[1030–1680 g]	(186 g) [1060–1449 g]	
Septum pellucidum			0.422**
Not available	1	1	
Intact	7	5	
Cavum/fenestrated	13	4	
CTE			NA
None	0	10	
Low stage	14	0	
High stage	7	0	

*CTE* chronic traumatic encephalopathy, *LOC* loss of consciousness, *NA* not assessed as data insufficient and/or inappropriate for analysis, *TBI* traumatic brain injury. *Student’s *t* test. **Fisher’s exact with data dichotomized as feature present versus absent

An acknowledged limitation of this study is that our case series represents a convenience sample of brains donated for research evaluation. Nevertheless, our observation that CTE pathology is present in around two-thirds of former rugby union players examined is in line with experience reporting neuropathological findings in other series of former contact sports athletes, including former American footballers and soccer players [7, 13]. Notably, a majority of our cases played solely at amateur level, including those with CTE. First played in the nineteenth century, rugby remained an amateur sport until 1995 when professionalism was permitted. With an average age among our cohort around 60 years, it is perhaps not surprising that the majority are defined as amateur. Intriguingly, some observers suggest that contact sport-related late adverse brain health outcomes might be restricted solely to professional athletes [5]. In respect of CTE, at least, our data suggest that level of participation does not protect against development of this neurodegenerative pathology.

In contrast, among this case series, there was clear association between length of rugby playing career and risk of CTE, which was independent of age at death. Again, this is in line with observations among several contact sports demonstrating longer playing careers associated with increased risk of a neurodegenerative disease diagnosis [3, 16] and, independently, with increased risk of CTE pathology [6, 10]. These data would be consistent with risk resulting from cumulative exposure to a factor associated with sport. To date, the only recognized risk factor for development of CTE is TBI and/or RHI exposure. Rugby union is recognized as having a notably high risk of concussion compared to wider contact sports [19], with risk at professional level increasing over the past 20 years [4].

In summary, in this convenience sample of research brain donations from former rugby union players, we found clear evidence of CTE pathology in around two-thirds of cases. Further, risk of CTE was directly associated with length of rugby playing career, interpreted as a surrogate for head impact exposure. These data reinforce concern around adverse brain health outcomes among former contact sports athletes and add to evidence in support of calls to reduce exposure to repetitive head impacts and risk of traumatic brain injury in training and in match play across all sports.

### Supplementary Information

Below is the link to the electronic supplementary material.

Supplementary file1 (DOCX 16 KB)

## Data Availability

The datasets supporting the conclusions of this article are available from participating archives on application.
