# Supplementary material for: Risk of chronic traumatic encephalopathy in rugby union is associated with length of playing career
Source: Acta Neuropathol. 2023 Oct 24;146(6):829–32. doi: 10.1007/s00401-023-02644-3 (PMC10627955; doi:10.1007/s00401-023-02644-3)
Supplement: Supplementary file 1 — Supplementary file1 (DOCX 16 KB) [file 401_2023_2644_MOESM1_ESM.docx]

|  | **CTE**  **N=21** | **No CTE**  **N=10** | **P*** |
| --- | --- | --- | --- |
| **Amyloid pathology**  None  Diffuse plaque  *Sparse*  *Moderate*  *Frequent*  Neuritic plaque  *Sparse*  *Moderate*  *Frequent*  Cerebral amyloid angiopathy | 9  11  *5*  *2*  *4*  9  *4*  *1*  *4*  8 | 4  6  *1*  *2*  *3*  5  *1*  *2*  *2*  4 | 1.000  1.000  1.000  1.000 |
| **LATE-NC**  None  Stage 1  Stage 2  Stage 3 | 14  1  4  2 | 8  1  1  - | 0.677 |
| **Lewy body pathology**  None  Present | 17  4 | 9  1 | 1.000 |
| **Cerebrovascular disease**  None  Chronic small vessel  Old infarction | 12  6  5 | 7  2  1 | 0.697 |
| **ARTAG**  None  Present | 12  9 | 9  1 | 0.106 |
| **Other neuropathology**  Primary age-related tauopathy  Corticobasal degeneration  Diffuse brain swelling with 2Y vascular complications  Glioblastoma  Cerebellar degeneration  Metastatic carcinoma | 1  0  1  1  0  0 | 0  1  1  0  1  0 | NA |

**Supplementary Table 1: Additional neuropathologies observed among former rugby players**

**ARTAG,** aging-related tau astrogliopathy; **CTE**, chronic traumatic encephalopathy; **LATE-NC**, Limbic-predominant age-related TDP-43 encephalopathy neuropathological change; **NA**, not assessed as data insufficient for analysis. ^*^Fisher’s exact with data dichotomized as feature present versus absent.
